# Reduced Graphene Oxide Functionalized with Cobalt Ferrite Nanocomposites for Enhanced Efficient and Lightweight Electromagnetic Wave Absorption

**DOI:** 10.1038/srep32381

**Published:** 2016-09-02

**Authors:** Yi Ding, Qingliang Liao, Shuo Liu, Huijing Guo, Yihui Sun, Guangjie Zhang, Yue Zhang

**Affiliations:** 1 State Key Laboratory for Advanced Metals and Materials, School of Materials Science and Engineering, University of Science and Technology Beijing, Beijing 100083, People’s Republic of China; 2 Beijing Municipal Key Laboratory of New Energy Materials and Technologies, University of Science and Technology Beijing, Beijing 100083, People’s Republic of China

## Abstract

In this paper, reduced graphene oxide functionalized with cobalt ferrite nanocomposites (CoFe@rGO) as a novel type of electromagnetic wave (EW) absorbing materials was successfully prepared by a three-step chemical method including hydrothermal synthesis, annealing process and mixing with paraffin. The effect of the sample thickness and the amount of paraffin on the EW absorption properties of the composites was studied, revealing that the absorption peaks shifted toward the low frequency regions with the increasing thickness while other conditions had little or no effect. It is found that the CoFe@rGO enhanced both dielectric losses and magnetic losses and had the best EW absorption properties and the wide wavelength coverage of the hole Ku-Band when adding only 5wt% composites to paraffin. Therefore, CoFe@rGO could be used as an efficient and lightweight EW absorber. Compared with the research into traditional absorbing materials, this figures of merit are typically of the same order of magnitude, but given the lightweight nature of the material and the high level of compatibility with mass production standards, making use of CoFe@rGO as an electromagnetic absorber material shows great potential for real product applications.

In recent years, electromagnetic wave (EW) absorbing materials have attracted substantial attention due to the widespread use in military and civil applications, such as electromagnetic protection, anechoic chambers, radiation-proof materials^1^,^2^,^3^. Following the microwave absorption mechanism, most EW absorbers are made of dielectric loss materials such as carbon nanotubes and conductive polymers, and magnetic loss materials, such as ferrite and ultrafine metal powders^4^,^5^,^6^,^7^,^8^,^9^,^10^,^11^. A lot of effort has been put into finding lightweight designs for the ideal EW absorbing materials with high absorption rate, wide absorption band, and reduced thickness^7^,^12^,^13^. Compared with traditional absorbing materials with magnetic loss as the main mechanism, the addition of dielectric loss materials can improve the EW absorption properties, broaden the absorption band and reduce the addictive amount, resulting in a lightweight and highly efficient absorbing material.

Metallic nano-oxides are an important type of absorbing materials; therefore, developing magnetic nano-materials with large magnetic loss and high magnetic permeability is a promising approach for absorbing materials^14^. Iron-based microstructured or nanostructured materials containing α-Fe_2_O_3_, γ-Fe_2_O_3_ and Fe_3_O_4_ have good EW absorption effect within a certain frequency range (millimeter waves, 2~18 GHz) due to their high magnetization intensity and magnetic anisotropy^15^,^16^,^17^. The operational frequency band of present satellite communications varies from 12–18 GHz (Ku band)^18^. It is difficult for a single nanoscale absorbing material to simultaneously achieve multi-band and wideband absorbance. Therefore research into nanostructured absorbing materials is focusing on nanocomposite absorbing materials. By adding the Co element into ferrite, the absorption performance of the prepared cobalt ferrite (CoFe) nanocomposite absorbing material can be significantly improved^19^,^20^,^21^. Ever since it’s successful isolation in 2004, graphene has become the research focus in the physics, chemistry, and materials science fields alike^22^,^23^,^24^. Graphene is a single atomic layer in a honeycomb two-dimensional structure; its hexagonal ring formed by sp^2^ hybridization of 3 carbon atoms with σ-bonds on the 2D plane and π-bonds orthogonal to them. Reduced graphene oxide (rGO), a widely-known derivative of graphene obtained from chemical reduction of graphene oxide by thermal, chemical or electrical treatments, possesses good dielectric properties, which can be used to prepare absorbing materials with dielectric loss mechanism^12^,^18^,^25^,^26^. The addition is of traditional absorbing material is usually more than 50%. Meanwhile, due to the addition of graphene, the additive amount to use of the type of absorber is greatly reduced.

Based on the above considerations, CoFe_2_O_4_ mixed to rGO nanocomposites (CoFe@rGO) were prepared by facile hydrothermal synthesis. It was found that when adding only 5 wt% composites to paraffin, the prepared CoFe@rGO with a thickness of 2.3 mm had the best EW absorption property. Compare with previous studies on the Fe_3_O_4_/graphene and CoFe_2_O_4_/graphene, this material had much lower amount (only 5%) and much wider bandwidth (7.17 GHz), which is much more suitable as a lightweight broadband electromagnetic wave absorbing coating. Considering its simple synthetic method and controllable ratio in the final composites, this kind of synthesized materials would be highly valuable in commercial use.

## Results

**Figure 1:**
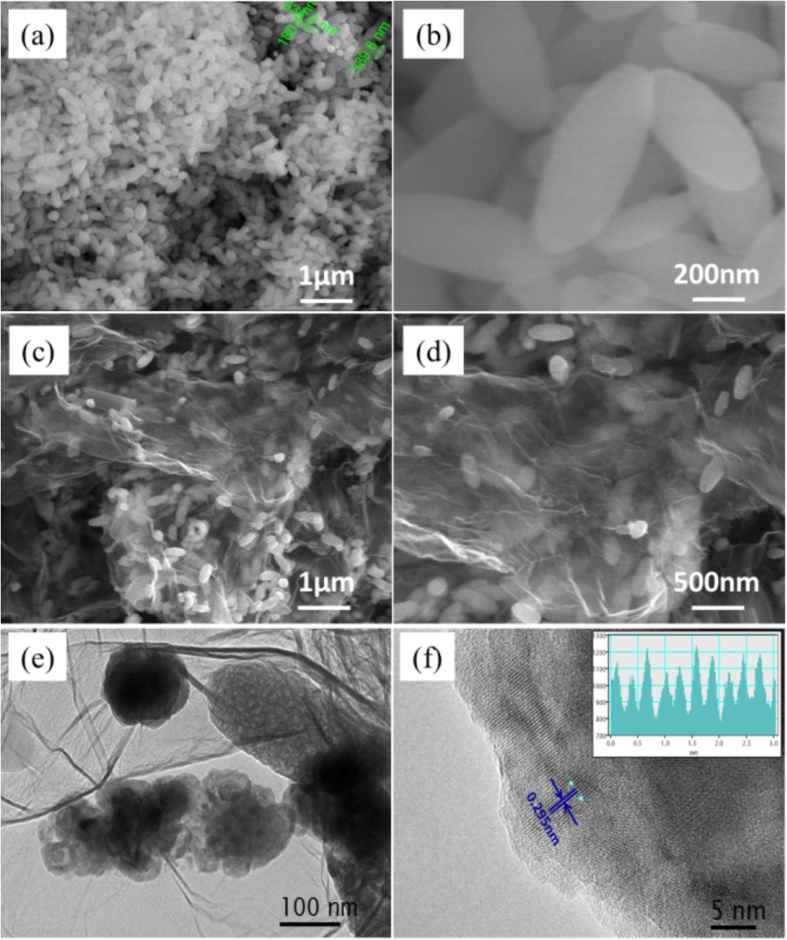
Typical SEM images of Co_x_Fe_2-x_O_3_ (**a**,**b**) and CoFe@rGO (**c**,**d**). Typical TEM images of CoFe@rGO (**e**,**f**).

Figure 1 includes SEM and TEM micrographs of the structure of the prepared Co_x_Fe_2-x_O_3_ and CoFe@rGO. Figure 1(a,b) shows that the prepared Co_x_Fe_2-x_O_3_ particles were short rod-like or rice-like shaped particles, densely packed together and exhibiting localized agglomeration. The rice-like particles were about 400 nm in length with a diameter of about 180 nm. Figure 1(c,d) shows the microstructure of CoFe@rGO, from which it can be seen that the crystal structure of the reduced graphene was in the shape of drape with transparent rough surface. Some drapes were stacked on the edge or surface. CoFe_2_O_4_ particles were unevenly distributed in the graphene substrate, presenting pronounced localized agglomeration. The rice-like particles were about 400 nm in length with a diameter of about 200 nm. Figure 1(e,f) shows the TEM image of CoFe@rGO, from which it can be seen that the CoFe_2_O_4_ are well-dispersed on RGO sheets. The high-resolution TEM image of the CoFe_2_O_4_ particles exhibits a lattice spacing of 0.295 nm corresponding to the (311) planes.

**Figure 2:**
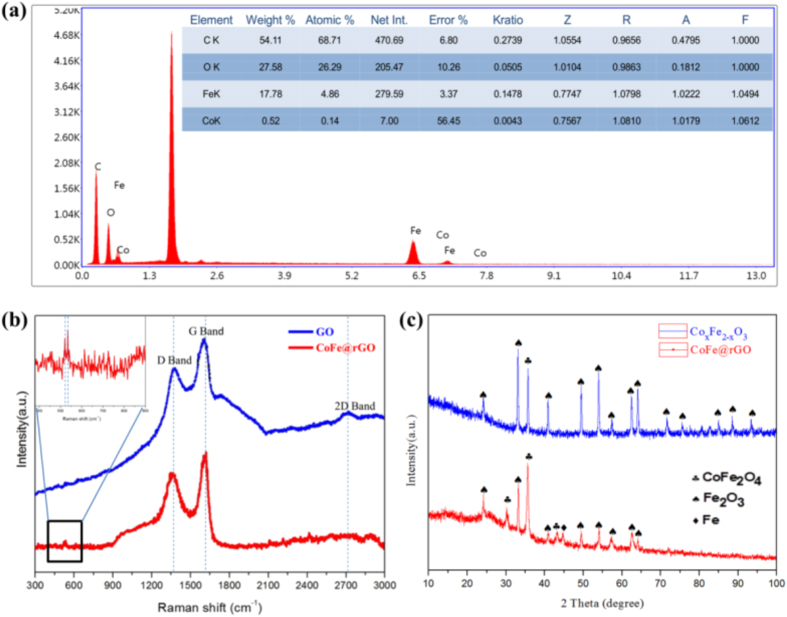
Structure characterization of CoFe_2_O_4_@rGO. (**a**) EDS pattern of CoFe@rGO; (**b**) Raman spectra of GO and CoFe@rGO; (**c**) X-ray diffraction pattern of CoFe@rGO.

Figure 2(a) shows the Energy Dispersive Spectrometer (EDS) analysis of the CoFe@rGO (the red square in Fig. 1(d)), which is proof of the presence of Co, Fe, O and C elementals in the composite. Raman spectrum analysis was carried out for the CoFe@rGO. The structure of the CoFe@rGO was characterized by excitation at a 514 nm wavelength on a Raman spectrometer. The results are shown in Fig. 2(b). The blue curve is the GO Raman spectrum, presenting the D-band at 1352 cm^−1^ and G-band at 1593 cm^−1^, and 2D-band at 2670 cm^−1^. For graphene, the D-band and G-band are the Raman characteristic bands. The D-band was due to the random arrangement of graphite or induced by lattice defects of C atoms. The higher the intensity of the D-band, the more the lattice defects in the crystals. The G-band is due to the stretching vibration of C atoms in the sp^2^-hybridized plane (degenerate regional center E_2g_ mode) while the 2D-band is due to the secondary Raman scattering of regional boundary phonon. The intensity ratio of D-band to G-band, I_D_/I_G_, could characterize the degree of crystal disorder. The intensity ratio of D-band to G-band (I_D_/I_G_) for graphene oxide was 0.88. The red curve represents the Raman spectrum for the prepared CoFe@rGO. The D-band, G-band and 2D-band of the CoFe@rGO were at 1345 cm^−1^, 1589 cm^−1^, and 2704 cm^−1^; the corresponding intensities were 86.26, 100.49, and 11.14. The ratio of D-band to G-band (I_D_/I_G_) was 0.86, slightly lower than that of the graphene oxide. This indicates that CoFe_2_O_4_ eliminated defects during the preparation of CoFe@GO nanocomposites, which affected the Raman peaks of graphene. There were weak Raman peaks for CoFe_2_O_4_ in the spectrum of CoFe@rGO at 520 cm^−1^, and 533 cm^−1^ in the inset of Fig. 2(b). This may be explained as follows: when preparing the composites, during the mixing of cobalt ferrite and graphene oxide by mass ratio 1:1, the volume of graphene oxide was large, while the volume of cobalt ferrite was relatively small. Therefore, it is more difficult to only find a part with cobalt ferrite for Raman spectroscopy analysis. Hence, it is difficult to reflect the presence of CoFe_2_O_4_ in the Raman spectrum.

Figure 2(c) presents the X-ray diffraction (XRD) analysis of the CoFe@rGO. Normally, the diffraction angle for graphene oxide is 10.4°, and the diffraction angle for the reduced graphene oxide is between 24° ~ 26°^27^. As can be seen from Fig. 2(b), no diffraction peaks appeared at 2θ = 10°. At about 24°, it is measured a diffraction peak with a relatively wide full width at half maximum (FWHM) in the red XRD curve of CoFe@rGO. And it is also measured in the blue XRD curve of Co_x_Fe_2-x_O_3_. This may be due to the overlap of the diffraction peak of the reduced graphene oxide and that of the ferric oxide, which indicates that during the heating process and annealing process of the facile hydrothermal synthesis, the ordered graphite crystal structure was reduced to some extent, that is, the graphene oxide was reduced into reduced graphene oxide. Moreover, apart from the reduced graphene oxide in the composites, we can detect the presence of CoFe_2_O_4_, Fe_2_O_3_, and Fe. The diffraction peaks of CoFe_2_O_4_ were at 30.08°, 35.24°, and 43.06°, corresponding to the crystal planes (220), (311), and (400). These peaks matched the standard PDF CARDS of CoFe_2_O_4_^28^. The diffraction peaks of Fe_2_O_3_ were found at 24.14°, 33.15°, 40.85°, 49.48°, 54.09°, 57.43°, 62.45°, and 63.99°; these peaks corresponded to the crystal plane reflection of the (012), (104), (113), (024), (116), (122), (214), and (300) planes respectively. The diffraction peaks of Fe_2_O_3_ matched the standard PDF CARDS^29^. The diffraction peak of Fe was found at 44.67°, corresponding to the crystal plane (110). This indicates that during annealing in the mixed gas atmosphere of H_2_/(H_2_ + Ar) = 8/100 at 360 °C for 3 h, CoFe_2_O_4_ and Fe were generated by reducing the cobalt ferrite. There were also diffraction peaks identifiable with the presence of Fe_2_O_3_ in the XRD result, implying that the cobalt ferrite was not fully reduced. The remaining cobalt ferrites existed as Co_x_Fe_2-x_O_3_ in the CoFe@rGO.

### Characterization of CoFe@rGO

To clarify the microwave absorption properties, the reflection loss (RL) can be calculated according to transmission line theory and using the relative complex permittivity and permeability^30^:


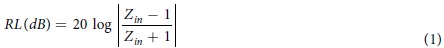


While the normalized input impedance (*Z*_*in*_) is calculated by:


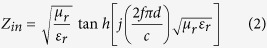


where, *f* is the frequency of electromagnetic waves, *c* is the velocity of electromagnetic waves in free space, and *d* is the thickness of the absorber, εr is the permittivity and μr is the permeability.

**Figure 3:**
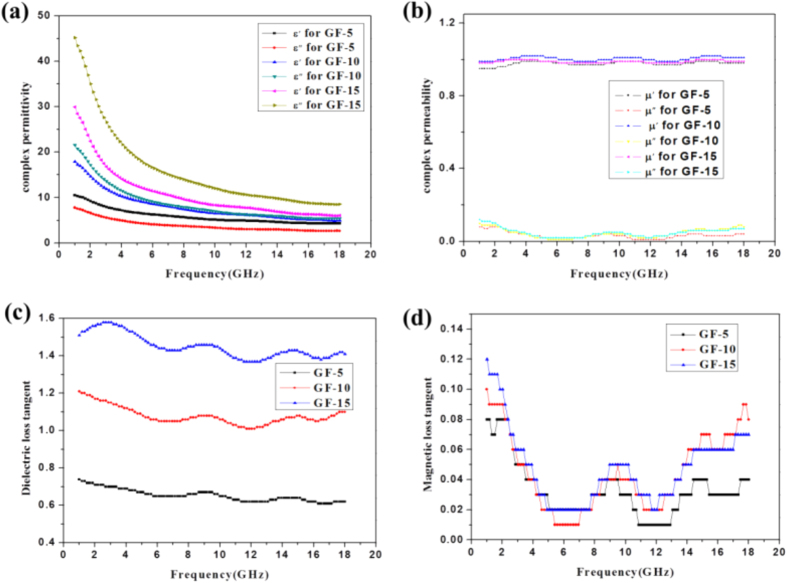
Dielectric characteristics of CoFe@rGO in the range of 2–18 GHz: (**a**) real part of permittivity, (**b**) imaginary part of permittivity, and (**c**) dielectric loss tangent and (**d**) magnetic loss tangent.

An automatic vector network parameter sweep-frequency measurement system HP-8722ES was used. The electromagnetic parameters of the composite materials with filling amount of 5 wt%, 10 wt% and 15 wt% (respectively, samples no. GF-5, GF-10, and GF-15) were measured by the coaxial reflection - transmission network method. The results are presented in Fig. 3. Figure 3(a) represents the complex permittivity of the GF-5, GF-10 and GF-15 samples, while Fig. 3(b) represents the complex permeability of the three composites. By comparing these curves, it is found that when decreasing the addition of CoFe@rGO from 15 wt% to 10 wt%, and further to 5 wt% to paraffin, both the real and the imaginary part of the complex permittivity were significantly reduced. With the increasing frequency of the EW, the real part of the complex permittivity of the sample GF-15 decreased from 29.91 to 6.03, while the imaginary part changed in the range of 8.52~45.17. For sample GF-5, the real part of the complex permittivity decreased from 10.51 to 4.29, while the imaginary part changed in the range of 2.66~7.76. Clearly, both the real part and the imaginary part of the complex permittivity decreased with the decreasing additive amount, indicating that the complex permittivity of the composite material can be controllable by varying the additive amount to paraffin, thereby influencing the absorption properties. The complex permeability of the three composites were barely changed with varying the amount of CoFe@rGO to paraffin. Both the real part and the imaginary part fluctuated to a limited extent. If the complex permittivity was too high, the complex permeability would be relatively low, leading to degradation of the impedance matching performance of the composites. Thereby, the EW absorption properties would be reduced.

The complex permittivity of GF-15 was too high, leading to poor matching with the complex permeability, such that the EW absorption properties had not been improved. For GF-5, its complex permeability was similar to that of GF-15, whereas the complex permittivity was obviously reduced, which is promising to improve the impedance matching characteristics of the composites. Therefore, the EW absorption properties of CoFe@rGO were enhanced.

According to the following equations:


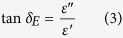



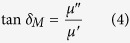


and using the eletromagnetic parameters of samples GF-5, GF-10 and GF-15, the dielectric loss tangent and the magnetic loss tangent were calculated. The relationship of the dielectric loss tangent vs. frequency and the magnetic loss tangent vs. frequency were plotted, as shown in Fig. 3(c,d). Figure 3(c,d) respectively show the dielectric and magnetic loss tangents of samples GF-5, GF-10 and GF-15. Figure 3(c) shows that when decreasing the addition of CoFe@rGO to paraffin, the dielectric loss tangent gradually decreased. With the increasing frequency of the EW, the dielectric loss tangent of GF-5, GF-10 and GF-15 fluctuated between 0.62 to 0.74, 1.10 to 1.21 and 1.41 to 1.51, respectively. It is concluded that the dielectric loss tangent decreased with increasing the additive amount to paraffin. Figure 3(d) shows that the magnetic loss tangent of GF-15 was the smallest. Compare with the EW absorption parameters of Fe_3_O_4_, the dielectric loss is increased and the magnetic loss is reduced with the inclusion of Co element into ferrite. In the frequency range of 2~14 GHz, with increasing the amount of CoFe@rGO to paraffin, the magnetic loss tangent gradually increased. Compared with samples GF-5 and GF-10, the magnetic loss tangent of sample GF-15 was improved in the range of 2~18 GHz, exhibiting a higher magnetic loss. Therefore, the impedance matching characteristics of the composites could be improved by carefuly controlling the addition of CoFe@rGO to paraffin.

**Figure 4:**
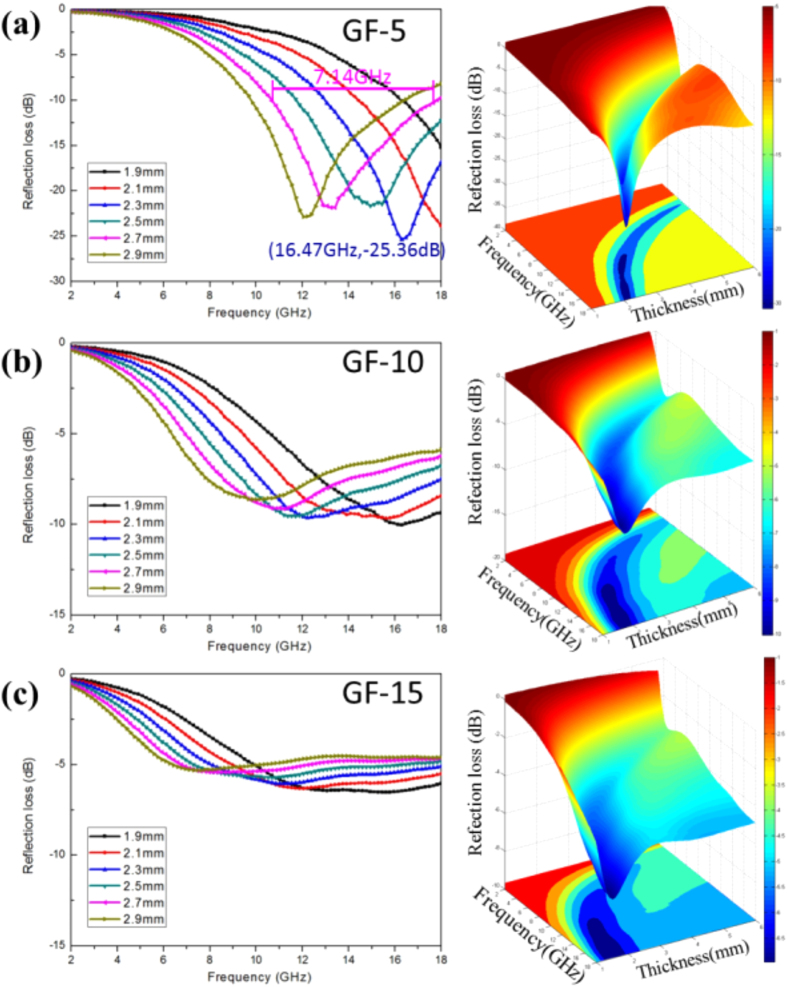
RL curves and 3D plots of CoFe@rGO at thicknesses ranging from 1 to 6 mm in the frequency range 2−18 GHz: (**a**) GF-5, (**b**) GF-10, and (**c**) GF-15.

Figure 4 presents the EW absorption properties of GF-5, GF-10, and GF-15 with different thicknesses calculated by transmission line theory. The specific EW absorption properties of GF-5 with different thicknesses are listed in Table 1. It can be seen that when the thickness was increased, the minimum peak of the reflection rate shifted to a low-frequency range. When the thickness was 2.3 mm, the corresponding frequency of the minimum peak of the reflection rate was 16.63 GHz. When the thickness increased to 3.3 mm, the corresponding frequency of the minimum reflection rate shifted to 10.86 GHz. The EW absorption property was the best when the sample thickness was 2.3 mm, in which, the reflection rate reached the lowest values of 16.63 GHz by −25.66 dB. Due to the frequencies measured in the test being in the radar wave frequency range, it was not possible to obtain any bandwidth corresponding to RL less than −10 dB. When the thickness was 2.7 mm, the bandwidth corresponding to RL less than −10 dB reached 7.17 GHz from 10.87 to 18.04 GHz. In this case, the minimum peak of the reflection rate of GF-5 was −21.64 dB, with good absorption property. Figure 4(b,c) show that for samples GF-10 and GF-15, with the increasing thickness, the minimum peak of the reflection rate shifted to the low-frequency range. However, the minimum reflection rates of the two samples were both above −10 dB, demonstrating poor EW absorption property compared with sample GF-5.

The EW absorption properties of CoFe@Rgo (GF-5, GF-10, and GF-15) were tabulated in Table 2. From Table 2, the sample GF-5 had obviously better performance than the other two. That is to say, when the addition of CoFe@rGO to paraffin was reduced to 5 wt%, the EW absorber was improved. The EW absorption of GF-5 was optimal when the thickness was 2.3 mm; the corresponding minimum peak of the reflection rate was −25.66 dB. When the thickness was 3.3 mm, the corresponding minimum peak of the reflection rate of GF-5 was −21.64 dB. For GF-10, the best EW absorption property was achieved when the thickness was 1.9 mm, and the corresponding minimum peak of the reflection rate was −10.10 dB. For GF-15, the best EW absorption property was achieved when the thickness was 1.6 mm, and the corresponding minimum peak of the reflection rate was −6.99 dB. The minimum reflection rates RL of GF-10 and GF-15 were both above −10 dB, presenting poor EW absorption property. Therefore, when the mass ratio of GO and CoFe_2_O_4_ was 1:1, after annealing in the mixed gas of H_2_/Ar for 3 h, the prepared CoFe@rGO by adding 5 wt% composites into paraffin had the best EW absorption property.

**Figure 5:**
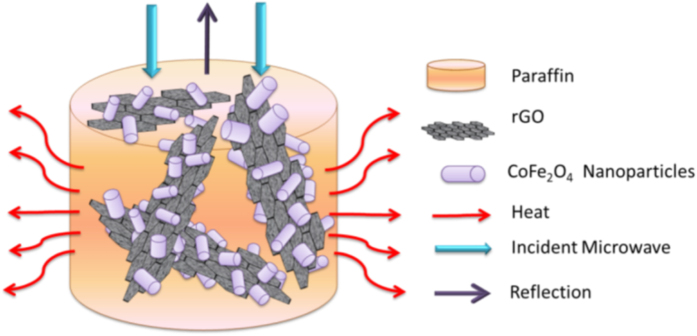
Schematic illustration showing how the microwave dissipated in three-dimensional network of CoFe@rGO.

According to the EW absorption properties of CoFe@rGO with different additive amounts, it is found that the complex permeability of GF-5, GF-10 and GF-15 were almost the same, whereas there were big differences in complex permittivity. GR-5 had the highest complex permittivity and showed the best EW absorption property, indicating that the additive amount of composites to paraffin could influence the dielectric loss significantly (shown in Fig. 5). However, it does not mean that the EW absorption property would become better with increasing the additive amount. The influence of impedance matching characteristics should be taken into consideration. Another thing that needs to be mentioned is that the dielectric loss tangent was far higher than the magnetic loss tangent. Therefore, the consumption of EW by composites mainly dependeds on the dielectric loss, which was essentially attributed to the polarization relaxation of rGO and the magnetic loss of the magnetic CoFe_2_O_4_ particles. For sure, the contribution of CoFe_2_O_4_ to the impedance matching of the composites and the EW absorption cannot be neglected. The microstructure of the composites also greatly promoted the absorption of the incident wave. In addition, other factors such as interface scattering and the polarization were as well beneficial to EW absorption. As a dielectric loss material, rGO was the main absorbent for enhancing the dielectric permittivity. The EW was absorbed by interacting with the electromagnetic field, while its degradation depended on the dielectric relaxation and interface polarization. When the EW entered into the composites, the directional movement of the carriers in the reduced graphene oxide formed a dispersion current, leading to dielectric relaxation and polarization at the interface. Thereby, the electromagnetic energy was consumed by being converted into heat. Meanwhile, the Fe in the composites acted as a dielectric material and could also absorb the EW by the polarization relaxation effect. By adding magnetic CoFe_2_O_4_ particles into the rGO dielectric material, the composites were formed. On the one hand, the impedance matching of the composites was improved. The impedance difference between the composites and air was reduced, which was beneficial to letting more EW into the inside of the composites. On the other hand, the composites were magnetized, generating natural resonance etc. The composites had a dual nature of magnetic and dielectric loss, which was conducive to the EW absorption and to broaden the absorption bandwidth. The interface polarization in the composites could facilitate the electromagnetic wave absorption. rGO had the characteristics of large specific surface area, layered structure and so on. When mixing with CoFe_2_O_4_ particles, a large number of interfaces were generated, like the interface between CoFe_2_O_4_ particles and rGO. The scattering and the polarization at the interface could induce interaction between electromagnetic waves, thereby facilitating the EW absorption by the composites.

## Discussion

In summary, a novel absorbing material reduced graphene oxide functionalized with cobalt ferrite nanocomposites (CoFe@rGO) was prepared. When the additive amount was 5 wt% to paraffin, the sample showed the best EW absorption property. In this case, when the thickness was 2.3 mm, the reflection rate reached the lowest at 16.63 GHz by −25.66 dB, and the bandwidth corresponding to RL less than −20 dB was 2.03 GHz. When the thickness was 2.7 mm, the bandwidth corresponding to RL less than −10 dB was 7.17 GHz from 10.87 to 18.04 GHz, covering the entire Ku band. The EW absorption mechanisms of CoFe@rGO were studied. The EW absorber was mainly attributed to the dielectric relaxation and polarization of rGO. Besides, the interface scattering and the hysteresis loss of CoFe_2_O_4_ contributed primarily to the EW absorption. The good absorbing property of the composites was due to the dielectric loss of CoFe_2_O_4_ and the dielectric relaxation and polarization of rGO. This novel and lightweight absorbing material has good prospects for commercial applications.

## Methods

CoFe@rGO were prepared by facile hydrothermal synthesis. The preparation process had three steps:

- Preparation of Co_x_Fe_2-x_O_3_ by hydrothermal reaction. Existing literature indicated α-Fe_2_O_3_ nano rings could be prepared by hydrothermal reaction by adding FeCl_3_. In a similar manner, the hydrothermal reaction for preparing Co_x_Fe_2-x_O_3_ was undertaken for 12 h at a furnace temperature of 220 °C by controlling the mole fraction of Co and Fe and adjusting the quality of FeCl_3_•6H_2_O and CoCl_2_•6H_2_O. After the reaction was completed, the reactor was cooled to room temperature in air. The solution was then centrifuged, and the precipitates were dried, giving the brick-red solid powder intermediate CoxFe_2-x_O_3_;
-   Preparation of CoFe_2_O_3_/rGO nanocomposites by facile hydrothermal synthesis with the prepared Co_x_Fe_2-x_O_3_ and graphene oxide (GO). The literature showed that graphene oxide GO could turn into reduced graphene rGO by hydrothermal reaction^31^. The GO and Co_x_Fe_2-x_O_3_ (mass ratio of 1:1) were uniformly dispersed in a solution of ethanol and glycerol (ratio of 3:1), followed by placing the mixed solution in the reactor for hydrothermal reaction at a furnace temperature of 180 °C for 12 h. After the reaction was completed, the reactor was cooled to room temperature in air. The solution was then centrifuged, and the precipitates were dried, giving brown Co_x_Fe_2-x_O_3_/rGO nanocomposites;
-  Preparation of CoFe@rGO. The prepared Co_x_Fe_2-x_O_3_/rGO nanocomposites from step (ii) were annealed at 360 °C in mixed gas of H_2_/Ar = 8/92. Finally, Co_x_Fe_2-x_O_3_/rGO nanocomposites were reduced into CoFe@rGO.

The different mixture proportions of the measured samples are illustrated in Table 3. GF-5, GF-10, and GF-15 were prepared with different contents of functionalized material to paraffin.

The samples were characterized by field emission scanning electron microscopy (FESEM) (FEI, Quanta 3D FEG), X-ray diffraction (XRD) (Rigaku DMAX-RB), and Raman spectroscopy (Jobin-Yvon, JY-HR800). The electromagnetic parameters of the samples were measured by a vector network analyzer system (HP722ES) from 2 to 18 GHz. Reflectivity loss values were calculated by Matlab software according to the complex permittivity and complex permeability. The CoFe@rGO prepared with the above process were mixed with paraffin to make a paste that could be shaped into coaxial samples. The electromagnetic parameters of the samples were tested by vector mesh analyzer to calculate the absorption properties. The sample had an inner diameter of 3.0 mm, an outer diameter of 7.0 mm, and a thickness of 2.0 mm. Three different sets of samples were prepared for comparative experiments, in which, the adding proportion of the composites were 5%, 10%, and 15%, respectively; while the amount of paraffin was 0.5 g for all three samples.

## Additional Information

**How to cite this article**: Ding, Y. *et al*. Reduced Graphene Oxide Functionalized with Cobalt Ferrite Nanocomposites for Enhanced Efficient and Lightweight Electromagnetic Wave Absorption. *Sci. Rep*. **6**, 32381; doi: 10.1038/srep32381 (2016).

## Figures and Tables

**Table 1:** The EW absorption properties of GF-5 with different thicknesses.

Thickness (mm)	RLmax (dB)	Absorption peak (GHz)	Bandwidth, Δf (RL < −10 dB) (GHz)	Corresponding bandwidth, Δf (RL < −10 dB) (GHz)
2.3	−25.66	16.63	4.67	13.24~18
2.5	−22.90	15.45	6.09	11.91~18
2.7	−21.64	13.41	7.17	10.87~18.04
2.9	−22.89	12.39	6.69	9.93~16.62
3.1	−22.33	11.54	6.18	9.16~15.34
3.3	−20.95	10.86	5.42	8.51~13.93

**Table 2:** The EW absorption properties of CoFe@rGO with different additive amounts (C denotes the added amount of composites to the paraffin matrix).

Sample	Reflection rate peak (dB)	C (wt%)	Corresponded thickness, (mm)	Bandwidth Δf (RL < −10 dB) (GHz)	Corresponding bandwidth Δf (RL < −10 dB) (GHz)	Reference
GF-5	−25.66	5	2.3	4.67	13.24~18	This work
GF-5	−21.64	5	2.7	7.17	10.87~18.04	This work
Fe_3_O_4_/carbon core/shell nanorods	−27.9	55	2	—	—	32
αFe_2_O_3_@CoFe_2_O_4_	−60	50	2	5	13~18	33
Carbonyls iron powders (CIPs)/Fe_3_O_4_	−38.1	60	2	6.3	8.7~15.0	15
rGO/Ni_0.4_Zn_0.4_Co_0.2_Fe_2_O_4_	−38.7		1.9	6.2	11.8~18	34
Fe_3_O_4_/SnO_2_	−27.38	80	4	—	—	35
SiO_2_@Fe_3_O_4_	−27.3	20	5	—	—	36

**Table 3:** Samples with different contents of functionalized material to paraffin.

Samples	GF-5	GF-10	GF-15
GO (wt%)	2.5	5	7.5
Co_x_Fe_2-x_O_3_ (wt%)	2.5	5	7.5

## References

[b1] Watts C. M. . Metamaterial electromagnetic wave absorbers. Adv Mater 24, OP98–120, OP181 (2012). 22627995 10.1002/adma.201200674

[b2] Micheli D. . Synthesis and electromagnetic characterization of frequency selective radar absorbing materials using carbon nanopowders. Carbon 77, 756–774 (2014).

[b3] Wang G. . High densities of magnetic nanoparticles supported on graphene fabricated by atomic layer deposition and their use as efficient synergistic microwave absorbers. Nano Research 7, 704–716 (2014).

[b4] Zhao T. . Electromagnetic wave absorbing properties of amorphous carbon nanotubes. Scientific Reports 4, 5619 (2014). 25007783 10.1038/srep05619 PMC4090627

[b5] Bhattacharya P. . Graphene and MWCNT based bi-functional polymer nanocomposites with enhanced microwave absorption and supercapacitor property. Materials Research Bulletin 66, 200–212 (2015).

[b6] Wang Z. & Zhao G.-L. Electromagnetic wave absorption of multi-walled carbon nanotube–epoxy composites in the R band. J. Mater. Chem. C 2, 9406–9411 (2014).

[b7] Shen B. . Lightweight, multifunctional polyetherimide/graphene@Fe_3_O_4_ composite foams for shielding of electromagnetic pollution. ACS Appl Mater Interfaces 5, 11383–11391 (2013). 24134429 10.1021/am4036527

[b8] Wang M. . Controlled synthesis and microwave absorption properties of Ni_0.6_Zn_0.4_Fe_2_O_4_/PANI composite via an *in-situ* polymerization process. Journal of Magnetism and Magnetic Materials 377, 52–58 (2015).

[b9] Wang W. . Wear-resistant and electromagnetic absorbing behaviors of oleic acid post-modified ferrite-filled epoxy resin composite coating. Journal of Magnetism and Magnetic Materials 378, 261–266 (2015).

[b10] Liu X. . Flexible nanocomposites with enhanced microwave absorption properties based on Fe_3_O_4_/SiO_2_ nanorods and polyvinylidene fluoride. J. Mater. Chem. A 3, 12197–12204 (2015).

[b11] Wang Y. . Synthesis and enhanced electromagnetic absorption properties of polypyrrole–BaFe_12_O_19_/Ni_0.8_Zn_0.2_Fe_2_O_4_ on graphene nanosheet. Synthetic Metals 196, 125–130 (2014).

[b12] Wu F. . Two-step reduction of self-assembed three-dimensional (3D) reduced graphene oxide (RGO)/zinc oxide (ZnO) nanocomposites for electromagnetic absorption. J. Mater. Chem. A 2, 20307–20315 (2014).

[b13] Liu W. W. . Fabrication of ultralight three-dimensional graphene networks with strong electromagnetic wave absorption properties. J Mater Chem A 3, 3739–3747 (2015).

[b14] Guan P. F. . Assembled Fe_3_O4 nanoparticles on graphene for enhanced electromagnetic wave losses. Applied Physics Letters 101, 153108 (2012).

[b15] Liu Q. . Enhanced microwave absorption properties of carbonyl iron/Fe_3_O_4_ composites synthesized by a simple hydrothermal method. Journal of Alloys and Compounds 561, 65–70 (2013).

[b16] Huo Y. . Controllable synthesis of hollow α-Fe_2_O_3_ nanostructures, their growth mechanism, and the morphology-reserved conversion to magnetic Fe_3_O_4_/C nanocomposites. RSC Advances 3, 19097 (2013).

[b17] Ren Y. . Growth of γ-Fe_2_O_3_ nanosheet arrays on graphene for electromagnetic absorption applications. RSC Advances 4, 21510 (2014).

[b18] Ding Y. . Electromagnetic wave absorption in reduced graphene oxide functionalized with Fe3O4/Fe nanorings. Nano Res. 9, 2018–2025 (2016).

[b19] Zeng M. . Electromagnetic Properties of Co/Co_3_O_4_/Reduced Graphene Oxide Nanocomposite. IEEE Transactions on Magnetics 50, 1–4 (2014).

[b20] Kong J. . Template-free synthesis of Co nanoporous structures and their electromagnetic wave absorption properties. Materials Letters 78, 69–71 (2012).

[b21] Xie S. . Carbon coated Co-SiC nanocomposite with high-performance microwave absorption. Physical chemistry chemical physics: PCCP 15, 16104–16110 (2013). 23986025 10.1039/c3cp52735b

[b22] Novoselov K. S. . A. Electric field effect in atomically thin carbon films. Science 306, 666–669 (2004). 15499015 10.1126/science.1102896

[b23] Geim A. K. & Novoselov K. S. The rise of graphene. Nature materials 6, 183–191 (2007). 17330084 10.1038/nmat1849

[b24] Wu J. . Graphenes as potential material for electronics. Chemical reviews 107, 718–747 (2007). 17291049 10.1021/cr068010r

[b25] Huang X. . Graphene-based composites. Chemical Society reviews 41, 666–686 (2012). 21796314 10.1039/c1cs15078b

[b26] Choi H. . Broadband electromagnetic response and ultrafast dynamics of few-layer epitaxial graphene. Applied Physics Letters 94, 172102 (2009).

[b27] Zong M. . One-pot hydrothermal synthesis of RGO/CoFe_2_O_4_ composite and its excellent microwave absorption properties. Materials Letters 114, 52–55 (2014).

[b28] Zhang S. . Vapor diffusion synthesis of rugby-shaped CoFe_2_O_4_/graphene composites as absorbing materials. Journal of Alloys and Compounds 630, 195–201 (2015).

[b29] Wang T. . Graphene–Fe_3_O_4_ nanohybrids: Synthesis and excellent electromagnetic absorption properties. Journal of Applied Physics 113, 024314 (2013).

[b30] Zhang L. . Investigation on the optimization, design and microwave absorption properties of reduced graphene oxide/tetrapod-like ZnO composites. RSC Adv. 5, 10197–10203 (2015).

[b31] Zhou J. . *In situ* controlled growth of ZnIn_2_S_4_ nanosheets on reduced graphene oxide for enhanced photocatalytic hydrogen production performance. Chem Commun 49, 2237–2239 (2013). 10.1039/c3cc38999e 23396572

[b32] Chen Y.-J. . Porous Fe_3_O_4_/Carbon Core/Shell Nanorods: Synthesis and Electromagnetic Properties. The Journal of Physical Chemistry C 115, 13603–13608 (2011).

[b33] Lv H. . Coin-like alpha-Fe2O3@CoFe_2_O_4_ core-shell composites with excellent electromagnetic absorption performance. ACS Appl Mater Interfaces 7, 4744–4750 (2015). 25664491 10.1021/am508438s

[b34] Liu P. . Preparation of reduced graphene oxide/Ni_0.4_Zn_0.4_Co_0.2_Fe_2_O_4_ nanocomposites and their excellent microwave absorption properties. Ceramics International 41, 13409–13416 (2015).

[b35] Chen Y.-J. . Porous Fe_3_O_4_/SnO_2_Core/Shell Nanorods: Synthesis and Electromagnetic Properties. The Journal of Physical Chemistry C 113, 10061–10064 (2009).

[b36] Ren Y. . Three-dimensional SiO_2_@Fe_3_O_4_ core/shell nanorod array/graphene architecture: synthesis and electromagnetic absorption properties. Nanoscale 5, 12296–12303 (2013). 24154630 10.1039/c3nr04058e

